# CHILDHOOD INTERPERSONAL TRAUMA AND DEPRESSION OF CHINESE IN MID LATER LIFE: MEDIATION EFFECT OF SOCIAL INTEGRATION

**DOI:** 10.1093/geroni/igad104.0613

**Published:** 2023-12-21

**Authors:** Jiajia Zhou, Shuai Zhou

**Affiliations:** The Hong Kong Polytechnic University, Hong Kong, Hong Kong; The Hong Kong Polytechnic University, Hong Kong, Hong Kong

## Abstract

**Objectives:**

This study investigates the long-term associations between five types of childhood interpersonal trauma (i.e., physical neglect, emotional neglect, physical abuse, being bullied, exposure to interparental violence) and depression in mid-later life. The mediation roles of social integration in the family (i.e., satisfaction with marriage and children) and broader social contexts (i.e., social participation and perceived social support) are examined in the associations.

**Methods:**

This study used two waves (2014 and 2015) of data from the China Health and Retirement Longitudinal Study. The analytical sample included 12,486 participants aged 45 years and older.

**Results:**

People who was bullied, physically abused, and witnessed interparental violence during childhood are more likely to develop depression in mid-later years, controlling for socio-demographic characteristics, childhood family socio-economic status, and childhood parental mental health. However, childhood physical neglect and emotional neglect are not directly related to depression in mid- and late-adulthood. The associations between childhood interpersonal trauma and depression were mediated by the satisfaction with marriage, satisfaction with children, and perceived social support, but not social engagement.

**Discussion:**

This study calls for attention on the long-term consequences of multiple types of childhood interpersonal trauma on mental health among middle-aged and older adults. It provides a comprehensive understanding of the mechanisms of social integration in bridging early-life interpersonal trauma and depression in mid-later life.

